# Predictive factors for somatization in a trauma sample

**DOI:** 10.1186/1745-0179-5-1

**Published:** 2009-01-06

**Authors:** Ask Elklit, Dorte M Christiansen

**Affiliations:** 1 Department of Psychology, University of Aarhus, Aarhus, Denmark

## Abstract

**Background:**

Unexplained somatic symptoms are common among trauma survivors. The relationship between trauma and somatization appears to be mediated by posttraumatic stress disorder (PTSD). However, only few studies have focused on what other psychological risk factors may predispose a trauma victim towards developing somatoform symptoms.

**Methods:**

The present paper examines the predictive value of PTSD severity, dissociation, negative affectivity, depression, anxiety, and feeling incompetent on somatization in a Danish sample of 169 adult men and women who were affected by a series of explosions in a firework factory settled in a residential area.

**Results:**

Negative affectivity and feelings of incompetence significantly predicted somatization, explaining 42% of the variance. PTSD was significant until negative affectivity was controlled for.

**Conclusion:**

Negative affectivity and feelings of incompetence significantly predicted somatization in the trauma sample whereas dissociation, depression, and anxiety were not associated with degree of somatization. PTSD as a risk factor was mediated by negative affectivity.

## Background

People exposed to trauma often suffer from a variety of psychological symptoms including anxiety, depression, and most importantly the psychiatric diagnoses of acute stress disorder (ASD) and posttraumatic stress disorder (PTSD). On top of this, many trauma types cause physical injuries that may cause lifelong suffering. However, even trauma victims that have not been seriously injured often report more somatic symptoms than do control groups not exposed to trauma. Such symptoms can be extremely disabling and are often a great source of psychological distress – partly due to the inability of health professionals to find any physical cause for the symptoms. Thus, the symptoms are often assumed to be caused by psychological processes and the patient is often dismissed by the health care system. However, the pain and suffering of such patients are real and whatever the cause, such symptoms are highly debilitating. Traditional medical treatment is often not effective in helping people with somatoform symptoms. In order to guide the search for more effective therapies, it is important to examine what causes these symptoms. In this article we want to examine the predictive effect of different potential risk factors on somatization in order to shed more light on what leads to unexplained somatic symptoms in trauma survivors.

### Somatization

Somatization refers to the development of somatic symptoms for which no organic cause is found [1,2]. Such symptoms are called somatoform. The DSM-IV [3] contains a diagnosis of somatization disorder which is given to people with a history of at least 8 different symptoms including at least four pain symptoms, two gastrointestinal symptoms, one sexual symptom and one pseudo neurological symptom not fully explained by a known general medical condition. The low prevalence of somatization in study samples has led researchers to use the less restrictive concept of abridged somatization, defined as the occurrence of at least 4 somatoform symptoms in men and 6 in women [4]. In contrast to this categorical approach, somatization is often considered to represent a continuum with few symptoms at one end and multiple symptoms relating to various body sites at the other [5]. Unless anything else is stated, throughout this article we will refer to somatization as a spectrum of somatoform symptoms of varying degrees.

### Somatization following trauma

Somatoform symptoms have consistently been linked to traumatic exposure. Trauma victims tend to score higher on self-reports of somatic complaints compared to controls [2,4,6-11]. It has been suggested that neurobiological changes, increased physiological arousal, and poorer health behaviour in the aftermath of trauma paves the way for somatization [12]. Furthermore, somatization may be related to other psychological consequences of trauma such as depression, anxiety, dissociation, and PTSD.

Van der Kolk, Pelcovitz, Roth, Mandel, McFarlane, and Herman [13] point out that somatoform symptoms, dissociation, and symptoms now present in the DSM-IV diagnosis of PTSD [3] were originally combined in the psychoanalytical concept of hysteria which was considered to be related to traumatic exposure [6]. They argue that the DSM-IV diagnosis of PTSD is too narrow to adequately capture all these symptoms which are more often than not coexisting in the aftermath of trauma. A similar point has been made by Brown, Cardeña, Nijenhuis, Sar, and Van der Hart [14]. In line with this view, several studies have found that this relationship between trauma and somatization is mediated by PTSD [2,11,15,16]. PTSD patients who report physical symptoms also report higher overall PTSD symptoms [6,17] and a higher frequency of depression [10,17] than PTSD patients who do not report such physical symptoms. The relation between PTSD and somatization may be explained by a lowered responsiveness towards external stimuli combined with an increased awareness of internal stimuli which has been found in people suffering from PTSD [11].

The correlation between PTSD and somatization does not tell us whether PTSD causes the somatic symptoms, whether the somatic symptoms cause PTSD, or whether the somatic symptoms and the PTSD symptomatology are both caused by a third variable. Andreski, Chilcoat, and Breslau [4] found that PTSD increased the risk for abridged somatization whereas the risk for new PTSD cases was not elevated in people with a history of abridged somatization. The Andreski et al. study thus supports the hypothesis that psychological stress caused by PTSD may increase personal vulnerability towards experiencing somatic symptoms.

### Risk factors

In a disaster study by North, Kawasaki, Spitznagel, and Hong [2], the prevalence of new somatoform symptoms following a traumatic event was found not to be associated with gender, injury or property damage. Also, no association was found between the number of physical symptoms and intensity of exposure to trauma [11]. Therefore, we have chosen to focus on posttraumatic and personality factors which may mediate the relationship between trauma and somatization.

#### PTSD symptom clusters

As mentioned, PTSD has repeatedly shown to be the most important predictor of somatization in trauma samples but it does not appear that the three symptom clusters of PTSD predict somatization equally well. McFarlane et al. [11] found that only the intrusion subscale achieved significance when using the different PTSD clusters to predict somatization. Intrusion may correlate with somatization because both are results of the disturbed information processing that is often present in PTSD, making it difficult for the victims to distinguish relevant from irrelevant information [11]. Contrary to this finding, Escalona, Achilles, Waitzkin, and Yager found PTSD numbing symptoms to be better at predicting somatization than the other avoidance symptoms as well as intrusion and arousal symptoms [1]. According to David Spiegel the numbing criteria defined by DSM-IV as a sense of isolation from others is consistent with a dissociated self-image [18]. Therefore these results may be due to dissociation increasing the risk of somatization.

#### Dissociation

It has been suggested that somatoform symptoms are caused by the dissociation of distressing material from conscious awareness caused by traumatic experiences in childhood [18]. More recently it has been suggested that physical symptoms in patients with PTSD may be a form of somatoform dissociation defined as the partial or complete loss of normal integration of somatoform components of experience, reactions, and functions [19]. Somatoform dissociation correlates highly with psychological dissociation and both are common in patients with PTSD [20]. In fact, dissociation has been suggested to be responsible for many of the most severe consequences of PTSD [21]. Patients with dissociative disorders as well as PTSD patients present more somatoform symptoms than other psychiatric patients [19,22]. Therefore, several researchers have investigated whether dissociation somehow mediates the relationship between PTSD and somatization.

Punamäki, Komproe, Quota, Elmasri, and de Jong found that peritraumatic dissociation did not have any mediating effect on the relationship between trauma and somatic symptoms [23]. In contrast, Salmon, Skaife, and Rhodes found that in patients with irritable bowel syndrome (IBS) persistent dissociation appeared as a mediating factor in the relationship between trauma and somatization [24]. It is thus possible that persistent but not peritraumatic dissociation predicts somatization. However, Salmon et al. did not control for PTSD and it is possible that if that had been done, it would have caused dissociation to lose significance [24].

#### Depression and anxiety

Somatization has been found to be related to high levels of psychological distress, anxiety and depressive diagnoses and functional impairment [5]. Previous studies have shown mood and anxiety disorders to be good predictors of somatization [25]. However, this may be due to the fact that many of these studies have not assessed PTSD. As both depression and anxiety disorders are frequently comorbid with PTSD, their correlation with somatization may be dependent on the relationship between PTSD and somatization.

Escalona et al. [1] studied women attending a primary care clinic at a department for Veteran Affairs and found that demographic variables as well as generalized anxiety disorder (GAD), panic disorder, and depression all failed to significantly predict somatization when PTSD was controlled for. Also, in a study of combat veterans by Beckham et al. [17] depression did not significantly predict number of somatic complaints. Contrary to this, other studies have found depression and anxiety to be significant predictors of somatization, even when PTSD is controlled for [2,11,26].

#### Negative affectivity

The overlapping constructs of negative affectivity and neuroticism are included in many factor models of personality including Costa and McCrae's five factor model of temperament where they are defined as the propensity to experience a wide variety of somatic and emotional dysphoric states including depression, anxiety, anger, and somatic symptoms [27]. People who score high on neuroticism are characterized by an inability to cope effectively with stress [28] and neuroticism and negative affectivity have been shown to play a role in the development of PTSD as well as other psychiatric disorders [7,29].

A part of the definition of negative affectivity is that it should make people more prone to experience somatoform symptoms. It is therefore not unexpected that several studies have shown neuroticism/negative affectivity to be implicated in somatization [4,5,27,28,30]. Negative affectivity has shown to correlate highest with non-specific symptoms such as stomach ache compared to more local and specific symptoms [31]. It has been suggested that neuroticism/negative affectivity serves as a risk factor for both PTSD and somatization [4] and that it may thus mediate the relationship between the two variables. However, to our knowledge, no study has examined the predictive value of negative affectivity on somatization specifically in a trauma sample and therefore it is not known how important negative affectivity is compared to trauma related factors such as PTSD and somatization.

#### Self-esteem/self-efficacy and related concepts

Studies have shown that people with PTSD often have lowered self-esteem. The causality in this relationship has not been very well studied but it probably goes both ways. Wong and Cook found that PTSD led to lower self-esteem and feelings of shame [32], whereas Adams and Boscanno found that low self-esteem significantly predicted PTSD [33]. The role of self-esteem and related concepts in somatization is not well examined either, but one study by Bödvarsdóttir and Elklit found that low self-worth was related to the development of somatic symptoms as well as PTSD following two Icelandic earthquakes [34]. The direction of the relationship, however, was not clear. In relation to this finding, Murphy found that self-efficacy significantly predicted somatization explaining 10% of the variance in survivors of the Mount St. Helens eruption [35]. Although these different concepts are not identical, the findings combined do suggest that being self-confident may be a protective factor, whereas being conscious of one self and one's body may heighten the risk for somatization.

## Methods

In the afternoon of November 3^rd ^2004 a series of explosions hit a firework factory in Seest, a suburb of the Danish city Kolding. One fireman was killed, about half a dozen residents were injured and 261 homes were partly or completely destroyed. The explosion measured 2.2 on the Richter scale and the costs of the disaster exceeded 100 million €. Most of the residents of the area were evacuated and many were unable to contact family members to make sure that they were safe. In average, people came into contact with their families after 2 1/2 hours but in one case family members were unable to come into contact with each other for three days. 51% of the sample had their home either partially or completely destroyed by the explosions. Those who still had a home returned after an average of 4 1/2 days. Further information has been published elsewhere [36].

### Procedures

PTSD, somatization and a number of other variables were measured at two time points. The first (T_1_) was three months after the accident and the second (T_2_) was one year later. Details of design and sampling have been accounted for elsewhere [36].

516 people (51% women, 49% men) participated in the study at T_1_. The data in the present study are from the 169 participants who answered the somatization questionnaire at both T_1 _and T_2_. Ages ranged from 18 to 95 years with a mean age of 50.2 years (SD = 14.7).

### Measures

• The Harvard Trauma Questionnaire part IV (HTQ) measures PTSD severity and estimates PTSD diagnosis according to the DSM-IV [37]. The HTQ contains 32 items based on the three subscales of PTSD concerning a potentially distressing event. The answers are scored on a four-point Likert scale ("not at all" (1), "a little" (2), "quite a bit (3), "all the time" (4)). The HTQ has good internal consistency, test-retest reliability and concurrent validity [37]. The alpha value for the total HTQ score was .93 in this study.

• The TSC was originally created by Briere and Runtz [38]. A Factor analysis has identified three subscales relating to somatization, negative affectivity and dissociation [39]. The somatization subscale consists of 8 items relating to headaches, stomach aches, respiratory problems and other non-specific somatic symptoms. Items are rated on a 4-point Likert scale ranging from "no" to "very often". The revised TSC has good reliability and good factor and criteria validity [39]. The alpha values in this study after three months were .82 for somatization, .85 for negative affectivity, and .63 for dissociation.

• The General Health Questionnaire-30 (GHQ-30) is based on the original 60 items edition of the GHQ [40]. In the GHQ-30 the somatic subscale has been removed and the items have been reduced to 30 [41]. The GHQ-30 therefore measures mainly psychological and psychosocial symptoms spread across five subscales measuring anxiety, feeling incompetent, depression, social dysfunction, and coping failure. Items are rated on a 4-point Likert scale rating from "a lot worse than usual", "worse than usual", "same as usual" to "better than usual". The sensitivity and specificity of the GHQ is estimated to be 81% and 80%, respectively [40]. The alpha value for the total GHQ-30 score in this study was .91. For depression the alpha value was .83, for anxiety it was .91, and for feeling incompetent it was .71.

### Statistics

The following results are based on somatization measured at T_2 _and the independent variables measured at T_1_. Mean values and standard deviations are given for each measure.

Multiple linear regression analyses were used to assess the predictive values of the different independent variables on somatization. When the predictive value of each measure had been established the significant values were entered into a regression analysis together in order to establish which values were still significant. A p-value of .05 was used to establish significance.

## Results

The mean score for somatization at T_2 _was 17.8 (SD = 5.6) ranging from 11 to 38. At T_1 _the mean total HTQ score was 51.4 (SD = 13.5). The participants scored highest on the avoidance subscale (M = 10.7, SD = 36), followed by the arousal subscale (M = 10.1, SD = 3.8), and the intrusion subscale (M = 9.3, SD = 3.0). It was estimated that approximately 13% of the participants met criteria for a PTSD diagnosis and 27% had sub-clinical PTSD, missing only one symptom in having a full PTSD diagnosis.

The sample that completed the questionnaire at both time points scored slightly higher on some of the measures than those who only completed the questionnaire at T1. Significant differences were found for negative affectivity, dissociation, reexperiencing, avoidance, and HTQ total score (all *p*s ≤ .05). No significant differences were found for gender, age, anxiety, depression, and feelings incompetence.

### PTSD

The three HTQ symptom clusters were entered into a linear regression analysis. Together they explained 33% of the variance but only the arousal factor was significant. When the two clusters of intrusion and avoidance were removed from the model, arousal alone explained 34% of the variance (*F *= 86.22, *p *≤ .005).

### Depression, anxiety, and feeling incompetent

At T_1 _the mean score on the depression subscale of the GHQ-30 was 7.8 (SD = 2.4). The mean score for anxiety was 16.4 (SD = 5.1), and for incompetence it was 12.5 (SD = 1.8). Depression, anxiety and incompetence all had significant and moderate correlations with somatization at T_2_(all *rho*'s ≥ .38, all *ps *≤ 0.001). All three variables were entered into a regression model but only anxiety and feeling incompetent made a significant contribution. Together these two risk factors accounted for 33% of the variance in somatization (*F *= 40.81, *p *≤ .05).

### Negative affectivity and dissociation

At T_1 _the mean score on dissociation was 6.1 (SD = 1.6) and the mean score on negative affectivity was 13.5 (SD = 3.8). Dissociation correlated moderately with somatization at T_2 _(*rho *= .40, *p *≤ 0.01) while the correlation between negative affectivity and somatization was high (*rho *= 0.62, *p *≤ .001). The dissociation and the negative affectivity subscales from the revised TSC were analyzed using linear regression. Alone, dissociation was significant, but it failed to remain so, when negative affectivity was introduced. Negative affectivity, however, was highly significant and explained 37% of the variance (*F *= 95.77, *p *≤ .005).

### Combination of significant risk factors

As a final step, all the significant measures were entered into a regression model together. When the risk factors were entered together only negative affectivity reached significance and feeling incompetent almost did (Table 1). We tested the predictive value of PTSD severity one last time by making a two-step regression analysis. At the first step we entered feelings of incompetence together with HTQ total score. They were both highly significant (both *ps *≤ .001) and together predicted 36% of the symptom variance. However, when negative affectivity entered the model at step two, HTQ total score completely lost significance leaving only feelings of incompetence and negative affectivity as significant predictors of somatization (both *ps *≤ .001). Together these two measures accounted for 42% of the total somatization variance (Table 2).

**Table 1:** significant risk factors.

Variable	Beta	Significance
HTQ arousal	.15	n.s.
GHQ anxiety	.09	n.s.
GHQ incompetence	.16	.053
TSC negative affectivity	.32	.002
CSS feeling let down	.06	n.s.

Note: Adjusted R square = .42

*F *= 22.73

n.s.: not significant

New regression analysis with the revised TSC somatization subscale as dependent variable and the previously significant measures as independent variables.

**Table 2:** Final model.

	Beta	Significance
GHQ incompetence	.27	≥ .001
TSC negative affectivity	.45	≥ .001

Note: Adjusted R square = .42

*F *= 57.44

Final regression analysis with the revised TSC somatization subscale as dependent variable and incompetence and negative affectivity as independent variables.

## Discussion

We did not in this sample find support for the hypothesis that dissociation and PTSD should be particularly related to somatization as suggested by Van der Kolk et al. [13]. Consistent with the finding by Salmon et al. [24], dissociation was a significant predictor of somatization but only until negative affectivity was controlled for. Even more surprisingly, and contrary to the findings from the studies mentioned in this article, PTSD did not emerge as the most important risk factor. In fact, PTSD severity failed entirely to significantly predict somatization after negative affectivity was controlled for.

Also contrary to previous findings, we found that arousal was the only PTSD symptom cluster to significantly predict somatization (although only until negative affectivity was controlled for). We did not examine numbing independently from the other avoidance symptoms, as was done in the Escalona et al. study, so we do not know whether numbing alone would have been significant. In relation to the other study mentioned earlier, McFarlane et al. used the IES to measure PTSD and thus did not measure arousal. However, they did point out that the cardiovascular, respiratory, and neurological symptoms that patients with PTSD often complain of are consistent with physical symptoms of arousal. Following this line of thought it should not be surprising that the arousal cluster in this study has proven to be a better predictor of somatization than both intrusion and avoidance/numbing. However, even the arousal cluster of the HTQ did not remain significant when negative affectivity and feeling incompetent were controlled for.

There are a few possible explanations for why PTSD and dissociation failed to ultimately predict somatization in this study. One possibility is that negative affectivity mediates the effect of PTSD on somatization. This hypothesis is supported by the fact that PTSD lost significance when negative affectivity was controlled for. However, another possibility is that the three concepts of dissociation, posttraumatic stress, and somatization are only connected following more intrusive traumas such as childhood sexual abuse or perhaps adult rape or torture. It is thus possible that both dissociation and PTSD would emerge as significant risk factors in such trauma samples even after controlling for negative affectivity. This hypothesis is supported by the finding that exposure to natural disasters (which has some features in common with the industrial disaster that the sample in the present study had been subjected to) tends to be associated with PTSD but to be less related to somatization, dissociation, and affect dysregulation than for example child abuse [13]. Last but not least, dissociation and PTSD were measured at three months in order to better establish a causal relationship between the two measures and somatization. However, if dissociation and PTSD are not just risk factors but part of the somatization process, somatization should correlate with dissociation and PTSD at T_2 _but not necessarily twelve months earlier. Therefore, the results do not show whether dissociation and PTSD are involved in the process of somatization but only that persistent psychological dissociation and symptoms of posttraumatic stress measured three months after an industrial accident affecting a residential area do not appear to explain the variance in somatization twelve months later above and beyond what can be explained by negative affectivity and feeling incompetent.

Depression and anxiety measured by the GHQ did not fare any better than PTSD and dissociation at predicting somatization. Whereas depression did not reach significance even when first entering the analysis, anxiety was originally significant but failed to remain so after feelings of incompetence, arousal, negative affectivity, and feeling let down were controlled for. This is probably due to negative affectivity mediating the relationship between anxiety and somatization. As the definition of negative affectivity is partly based on the tendency to experience fear and anxiety it is not surprising that there should be some overlap between the two concepts. It is intriguing that anxiety proved a better predictor of somatization than did depression. As mentioned earlier, studies have generally found depression to be a better predictor of somatization than anxiety. This unexpected finding may be related to the use of an instrument that is not specifically designed to measure the two variables.

In contrast to all these variables, feeling incompetent and negative affectivity did significantly predict somatization in this sample, together accounting for 42% of the somatoform symptom variance. As for feeling incompetent, it is quite interesting that a psychological measure that has been so little in the focus of research, actually proved better at predicting somatization than did otherwise well-established risk factors. What is really interesting is that feeling incompetent was actually the only single factor that remained significant when negative affectivity was controlled for. This is despite the fact that such a measure of low self-esteem/self-efficacy could well be hypothesized to be mediated by negative affectivity as well as by PTSD. As the GHQ measure was taken at T_1 _while somatization was measured at T_2_, these results suggest that feeling incompetent increases the risk of somatization, possibly by influencing the person's attempts to cope with the traumatic event as well as with any somatoform symptoms. According to Murphy et al. [35], whether a person engages in coping attempts depends on the expectations he or she has concerning their success and expecting failure may decrease the effect of any such coping attempts. However, this relationship probably works both ways, and somatization is very likely to further decrease self-esteem and lead to feelings of incompetence.

It is not as unexpected that we found negative affectivity to be highly predictive of somatization, as this is in line with studies on non-traumatised samples. However, it is very interesting that we found negative affectivity to mediate the effect of PTSD on somatization to such an extent that HTQ total score as well as the arousal sub-scale score failed to significantly predict somatization. As no other study to our knowledge has controlled for negative affectivity when examining somatization in a trauma sample, this important finding can neither be supported nor contradicted by other research.

It has been proposed that trait negative affectivity has a general non-specific relationship with symptom reporting, suggesting that people high on negative affectivity are interoceptively hypervigilant and thus notice bodily changes that go unnoticed in other people [31]. Another way through which negative affectivity may influence reporting of somatic symptoms is through recall bias caused by state-dependant recall [41]. Furthermore, neuroticism/negative affectivity may increase the actual prevalence of somatic symptoms through risk behaviours such as smoking, drinking, and using drugs [28]. Also, studies have shown that people who score high on neuroticism tend to have poor eating, sleeping, and exercise habits [28].

Negative affectivity is not uniquely associated with somatization but appears to be a general predictor of symptomatology. This is probably the reason why it appeared to mediate the effect of both PTSD, dissociation, and anxiety on somatization in this study. Furthermore, negative affectivity is related to somatization even in the absence of a traumatic stressor. This study therefore suggests that even though somatization is particularly prevalent in traumatised populations, the mechanisms behind traumatisation do not appear to differ between traumatised and general populations. (although it should be noted that a general population is not necessarily traumatised). Thus, even though PTSD and dissociation appear to be associated with degree of somatization in the aftermath of trauma, they do not surpass the importance of negative affectivity as a non-specific risk factor of somatization.

### Limitations of the study

There are several limitations to this study. Most importantly, the use of combined measures was made necessary by the high number of variables examined, as more thorough testing of each single variable would have made the questionnaire too time consuming for the participants. However, the TSC and the GHQ are designed to test several psychological constructs combined and may not measure variables such as dissociation and depression as thoroughly as an instrument designed specifically to test such variables. For example, it can be argued that the five items of the dissociation subscale used in this study is not quite enough to make a good estimate of dissociation in a traumatised sample. Furthermore, we assessed only persistent psychological dissociation. Thus, neither peritraumatic nor somatoform dissociation will be revealed using the revised TSC. Though persistent/pathological dissociation has been shown to correlate with peritraumatic dissociation [43] the two measures may not predict somatization equally well.

Another limitation is that many of the houses in the disaster area had to undergo major repairs and some had to be rebuilt completely. This is a process that takes very long time and for some people it was further delayed by problems with insurance companies [36]. This means that the general level of stress can be expected to be quite high due to practical issues, relocation, and insecurity concerning insurance outcomes. There is a risk that these stressors may have influenced some of the different measures. For example, negative affectivity is supposed to measure a personality trait, but the negative affectivity scores may have been increased by a high level of general stress in the sample.

Last but not least, the sample consisted of people from the same small area of similar ethnicity, cultural background, and middle class socioeconomic status. The results found here cannot automatically be extrapolated to populations from other countries and backgrounds exposed to different types of trauma.

### Future research

Many studies have established the role of PTSD as a mediating factor in the relationship between trauma and somatization. However, we found PTSD severity to completely lose significance when negative affectivity was controlled for. Several studies have shown negative affectivity to be implicated in somatization but it is surprising that it appears to be such a strong risk factor that it can even eliminate the effect of PTSD in a trauma sample. This study highlights negative affectivity as a variable not to be ignored when examining somatization. Particularly, negative affectivity should be controlled for when predicting somatization following the kind of trauma that is expected to result in higher degree of comorbidity between PTSD, dissociation, and somatization.

Also, feeling incompetent has not been very well studied in relation to trauma and somatization and it is interesting that it remains significant when otherwise well-established risk factors such as anxiety, depression, and even PTSD severity do not. More guided research focusing on variables such as self worth and self-efficacy will give more detailed information on how it affects somatization.

Finally, this study examined a sample where all participants had been subject to the same traumatic event. It should be studied whether the same risk factors apply to somatization after other trauma types – especially after more personal traumas such as torture, physical assault, or rape where victims for example tend to dissociate more.

## Conclusion

Contrary to what other studies have found, depression did not significantly predict somatization in this study and nor did anxiety and dissociation after negative affectivity was controlled for. Even more interesting, PTSD did not significantly predict somatization after controlling for negative affectivity, suggesting that negative affectivity is a more important predictor of somatization even in trauma samples than PTSD. In stead, negative affectivity and feelings of incompetence significantly predicted the degree of somatization, together accounting for 42% of the variance. This finding is to our knowledge unprecedented.

## Competing interests

The authors declare that they have no competing interests.

## Authors' contributions

AE carried out the studies, performed the statistical analyses, supervised the writing of the article and drafted the manuscript. DMC performed the statistical analyses and wrote the article. Both authors read and approved of the final manuscript.
